# Simulation and Experimental Study on the Surface Generation Mechanism of Cu Alloys in Ultra-Precision Diamond Turning

**DOI:** 10.3390/mi10090573

**Published:** 2019-08-29

**Authors:** Quanli Zhang, Nan Guo, Yan Chen, Yucan Fu, Qingliang Zhao

**Affiliations:** 1 Jiangsu Key Laboratory of Precision and Micro-Manufacturing Technology, Nanjing University of Aeronautics and Astronautics, Nanjing 210016, China; 2 College of Mechanical and Electrical Engineering, Nanjing University of Aeronautics and Astronautics, Nanjing 210016, China; 3 Centre for Precision Engineering, School of Mechatronics Engineering, Harbin Institute of Technology, Harbin 150001, China

**Keywords:** ultra-precision diamond turning, Cu alloy, modeling, tool wear

## Abstract

The surface generation mechanism of the Cu alloys in ultra-precision diamond turning is investigated by both simulation and experimental methods, where the effects of the cutting parameters on the surface characteristics are explored, including the workpiece spindle speed, the cutting depth, the feed rate and the nose radius of the diamond tool. To verify the built model, the cutting experiments are conducted at selected parameters, where the causes of the error between the simulation and the machining results are analyzed, including the effects of the materials microstructure and the diamond tool wear. In addition, the nanometric surface characteristics of the Cu alloys after the diamond turning are identified, including the finer scratching grooves caused by the tool wear, the formation of the surface burs and the adhesion of graphite. The results show that the built model can be basically used to predict the surface topography for the selection of the appropriate machining parameters in the ultra-precision diamond turning process.

## 1. Introduction

Ultra-precision diamond turning is widely used to manufacture copper (Cu), aluminum (Al) and even some brittle semi-conductor materials, such as monocrystalline silicon (Si) and germanium (Ge) [1]. With the development of tool servo techniques in recent years, the single point diamond turning (SPDT) method is widely used in fabricating high-quality free-form optical devices efficiently to achieve extremely high surface quality with the surface roughness *Sa* < 10 nm and the form accuracy *PV* < 0.1 μm [2,3,4], to meet the requirement of the high quality for medical facilities, automotive fields, military and aerospace industries. Actually, the material removal and the surface generation is a comprehensive process in ultra-precision diamond turning, which is affected by many factors, including the workpiece material, the machining parameters, the cutting tool conditions and the accuracy of the machine tool.

Simulation of the generated surface topography in the ultra-precision diamond turning process of easy-to-cut materials is widely investigated. Cheung and Lee used a microplasticity model to study the obtained surface topography of the single crystalline copper, which was dependent on the crystallographic orientation, and the simulated results were found to correlate well with the experimental results [5]. Recently, He and Zong proposed a quadratic distribution function to study the influence of the material elastic deformation and the plastic flow. The effects of the kinematics and the defects in the workpiece material were taken into consideration in the prediction model [6]. Specifically for the manufacturing of the freeform surfaces, Huang and Liang tried to use a more general simulation model for the machining process of the spherical and freedom surfaces [7]. Kong et al. used a model-based simulation system to predict the surface topography and analyze the effect of the motion errors on the surface generation in fast tool servo (FTS) machining [8]. Li et al. simulated the slow tool servo (STS) machining progress, where the tool path planning, the tool geometry selection and the tool radius compensation were studied, and the simulated result showed that a uniform surface topography could be obtained over the entire surface which agreed well with the real cutting conditions [9]. In addition, the surface characteristics are closely dependent on the geometry of the diamond tool. However, in the single point diamond turning process, the cutting tool does not keep its geometry due to the wear process caused by the high temperature and the fluctuating cutting force [10,11]. Sawangsri and Cheng [12] proposed a new model of the cutting force for ultra-precision and micro-cutting to analyze the micro-cutting mechanics and to detect the tool wear. Zhang and To [13] also reported that a characteristic frequency appeared for the power spectra analysis of the cutting force in the fly-cutting process, which can be adopted to monitor the diamond tool wear, and their further research showed that the tool wear characteristics could be identified by the first-order modal vibration [14]. In the wear mechanism, the graphitization of diamond is considered as a main factor for the tool wear in the diamond turning [15], which then results in the micro-chipping of the tool edge. As far as the nanoscale surface quality for the machined part is concerned, the effects of the chipping-induced irregularities on the tool nose on the machined surface could not be neglected, so the effects of the diamond tool wear should be identified.

For the surface analysis, the traditional characterization of the micro-roughness mainly focused on evaluating either the statistical characteristics or the extreme value characteristics of the roughness profile like surface roughness (*Ra*) or peak-to-valley height (*PV*) and cannot reflect the complexity of the surface profile. The tool feed, the spindle rotational errors, the tool geometry and the relative tool-work vibration will influence the surface roughness in diamond turning [16]. These components would appear at different frequencies in the surface roughness spectrum. The method of using power spectrum density (PSD) calculation can get data from different frequencies to better illustrate the surface formation mechanism during the ultra-precision diamond turning process.

Based on the above consideration, a comprehensive study on the surface generation mechanism in ultra-precision diamond turning of Cu alloys is undertaken in the present work, taking the effects of the machining parameters, the diamond tool wear, the relative vibration and the material microstructure into consideration.

## 2. Modeling of the Surface Topography

**Figure 1:**
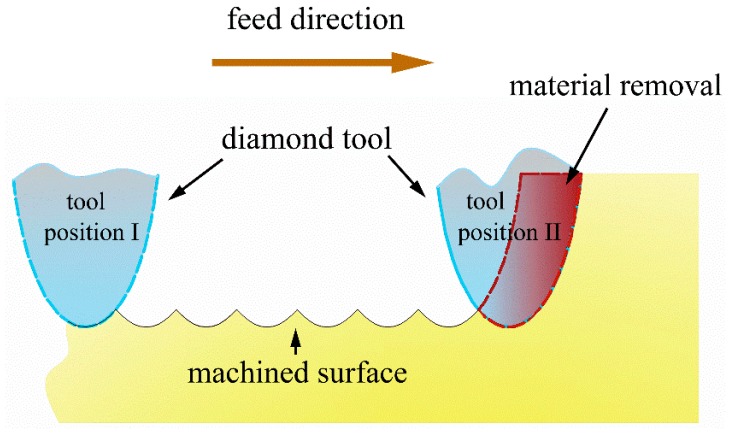
The cross-sectional profile generated in the ultra-precision diamond turning process under the ideal condition.

Under an ideal condition, without considering the material properties, the tool wear, the machine stability, etc., the surface topography of the workpiece after the machining can be regarded as the sweeping area of the cutting tool in a fixed trajectory. Therefore, the cross-sectional surface profile of the turned surface along the radial direction can be regarded as the repeated tool profiles at the intervals of the feed marks. As illustrated by Figure 1, when the diamond tool moves from position I to position II, the material is removed step by step. Under the ideal condition, the step material removal is the red area.

The maximum peak-to-valley height *PV* under the ideal conditions can be derived as follows,

(1) $$PV=rad_{t}-\sqrt{rad_{t}^{2}-\frac{{s_{s}}^{2}}{4}}=rad_{t}\cdot \left[1-\sqrt{1-\frac{{s_{s}}^{2}}{4\cdot rad_{t}^{2}}}\right]$$

for *s_s_* << *rad_t_*, we can get that,

(2) $$1-\sqrt{1-{s_{s}}^{2}/\left(4\cdot rad_{t}^{2}\right)}\approx {s_{s}}^{2}/\left(8\cdot rad_{t}^{2}\right)$$

so,

(3) $$PV=\frac{{s_{s}}^{2}}{8\cdot rad_{t}}$$

and the arithmetic roughness *R_a_* is expressed by Equation (4),

(4) $$R_{a}\approx \frac{0.032{s_{s}}^{2}}{rad_{t}}$$

where *s_s_* is the feed rate per revolution, *rad_t_* is the tool nose radius.

### 2.1. Principles of the 3D Surface Topography Simulation in Ultra-Precision Diamond Turning

From the 2-dimensional surface profile modeling, we cannot get the full information of the surface characteristics of the machined surface, so the simulation of the 3D surface topography is performed to describe the generated surface under the ideal conditions, and the surface data, including the surface roughness (*R_a_*, *R_t_*, *R_q_*), the form accuracy (*PV*) and the surface waviness, can be determined from the simulated results.

**Figure 2:**
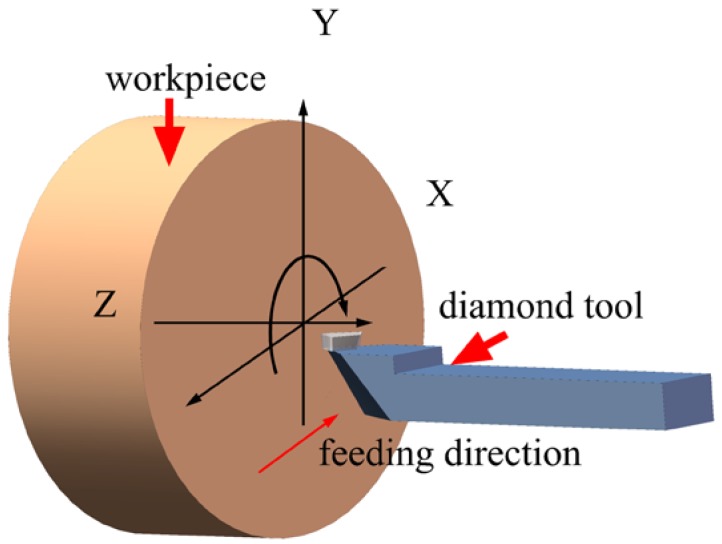
Schematic diagram of the ultra-precision diamond turning process, where the diamond tool moves from the edge towards the center of the workpiece.

**Figure 3:**
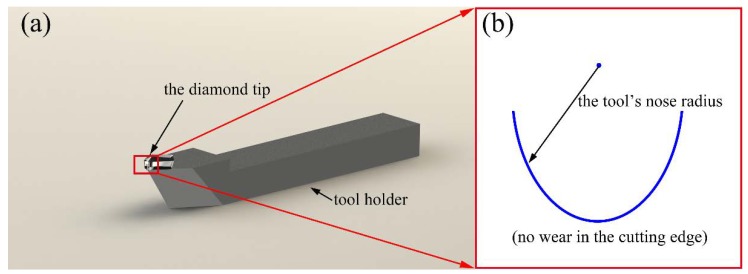
(**a**) The photograph of the used diamond tool, (**b**) the geometry of the diamond tool edge.

Figure 2 shows the schematic diagram of the single point diamond turning (SPDT) process. The diamond tool moves along the X-axis at a certain feed rate with the workpiece rotated around the Z-axis. During the cutting process, the diamond tool moves in a spiral locus from the edge to the center of the workpiece. The photograph and the geometric shape of the tool used in this study are shown in Figure 3.

**Figure 4:**
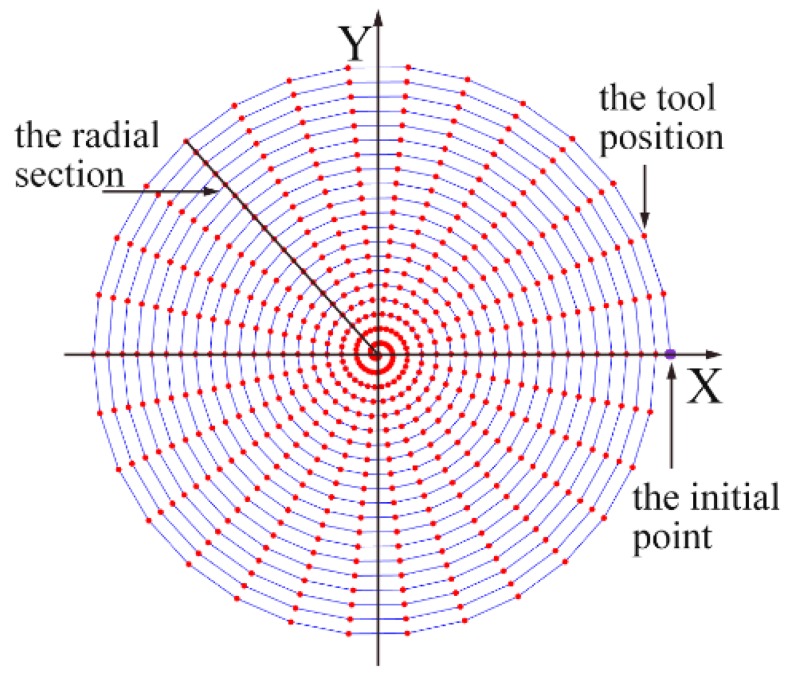
The spiral tool trajectory and the distributed tool positions in the modeling, where the red points denote different positions of the tool in the cutting process.

In the modeling process, the trajectory of the tool position in the machining process should be determined. Under a constant feed rate and a certain rotation speed of the workpiece, the infinite tool positions can be obtained, as shown in Figure 4, and each position of the tool can be expressed in the polar coordinates as:

(5) $$\begin{array}{l} R=R_{0}-t\times f_{s}\\ \theta =t\times \omega \end{array}$$

where *t* is the machining time, *f_s_* is the feed rate (mm/min), *ω* is the rotational speed (rad/s), *R* is the distance from the tool tip position to the center of the workpiece and *R*_0_ is the radius of the workpiece. In the model, the angle *θ* is kept changing until the machining is finished. Let *θ_i_* represent the *i*th position of the tool in the trajectory, and the corresponding coordinates in the X-Y plane can be expressed as,

(6) $$\begin{array}{l} X_{i}(t)=R\sin\left(\theta_{i}\right)=\left(R_{0}-f_{s}\cdot \theta_{i}/\omega \right)\cdot \sin\left(\theta_{i}\right)\\ Y_{i}(t)=R\cos\left(\theta_{i}\right)=\left(R_{0}-f_{s}\cdot \theta_{i}/\omega \right)\cdot \cos\left(\theta_{i}\right) \end{array}.$$

The simulation of the surface topography of the Cu alloy in ultra-precision diamond turning is calculated by MATLAB with the following steps:

- Firstly, the length and width of the workpiece are input as initial parameters, and the resolution is defined with the height data set at every point at the initial value.
- Secondly, the tool is moved along the calculated spiral trajectory. When the tool arrives at the fixed point in the trajectory, the height of the workpiece within a small area under the cutting tool is compared with the lower surface of the tool. If the area is higher than the lower surface of the tool, the area will be removed and the geometry of the tool is copied to the machined surface.
- Finally, when the cutting tool reaches the center of the workpiece, the simulation is completed, and the surface heights of the points we set in the first step can be obtained.

**Figure 5:**
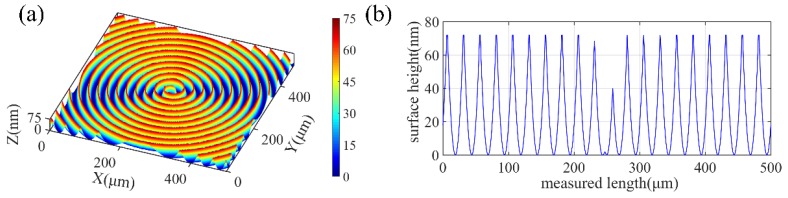
One of the typical simulated surfaces under an ideal condition. Here, (**a**) is a typical 3D surface topography and (**b**) is a cross-sectional profile from (**a**).

One of the typical simulated surfaces is shown in Figure 5a, and the cross-sectional profile of the simulated surface along the radial direction is also extracted, which is shown in Figure 5b.

As far as the simulated surface profile is collected, the information of the surface roughness and the form accuracy can be obtained based on the following equations,

(7) $$R_{a}=\frac{1}{n}\sum_{i=1}^{n}\left|z_{j}-z_{mean}\right|,$$

(8) $$PV=\max\left\{z_{j}\right\}-\min\left\{z_{j}\right\},$$

where *z_j_* is the *j*th simulated surface profile height on the cross lattice and *z_mean_* is the mean height along the profile.

### 2.2. Simulation of the 3D Surface Topography at Different Machining Parameters

Once the simulation model is built, the 3D surface topography under different cutting parameters can be achieved, and the surface roughness and the form accuracy are extracted to evaluate the surface quality machined by the ultra-precision diamond cutting process at the different cutting depth, the different feed rate, the different main spindle speed and the different tool nose radius. The cutting parameters and the tool nose radius set in this study are listed in Table 1.

**Figure 6:**
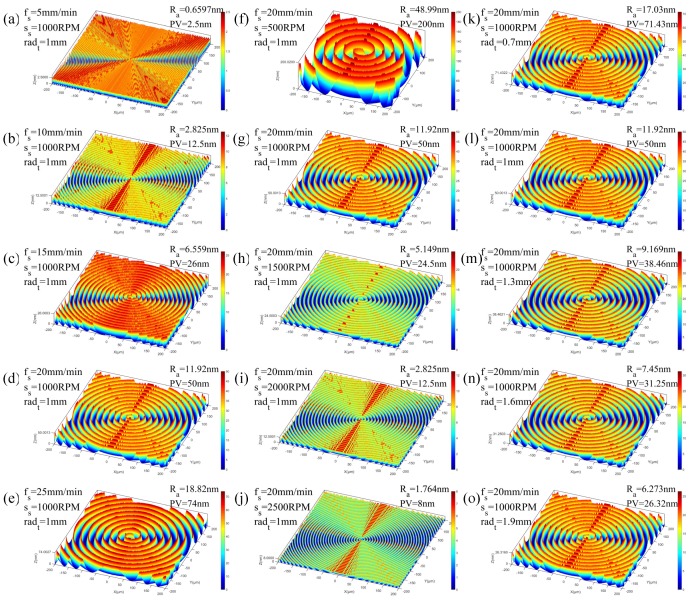
The simulated 3D surface topographies under different cutting parameters. Here, (**a**–**e**) are for the varying feed speed, (**f**–**j**) are for the varying spindle speed, and (**k**–**o**) are for the varying tool nose radius.

The simulated surface topography of the diamond turned Cu alloys are shown in Figure 6. In the figure, *f_s_* is the feed rate, *s_s_* is the workpiece spindle speed and *rad_t_* is the tool nose radius. The corresponding surface roughness *R_a_* and the form accuracy (*PV*) are also labeled in the figure.

**Figure 7:**
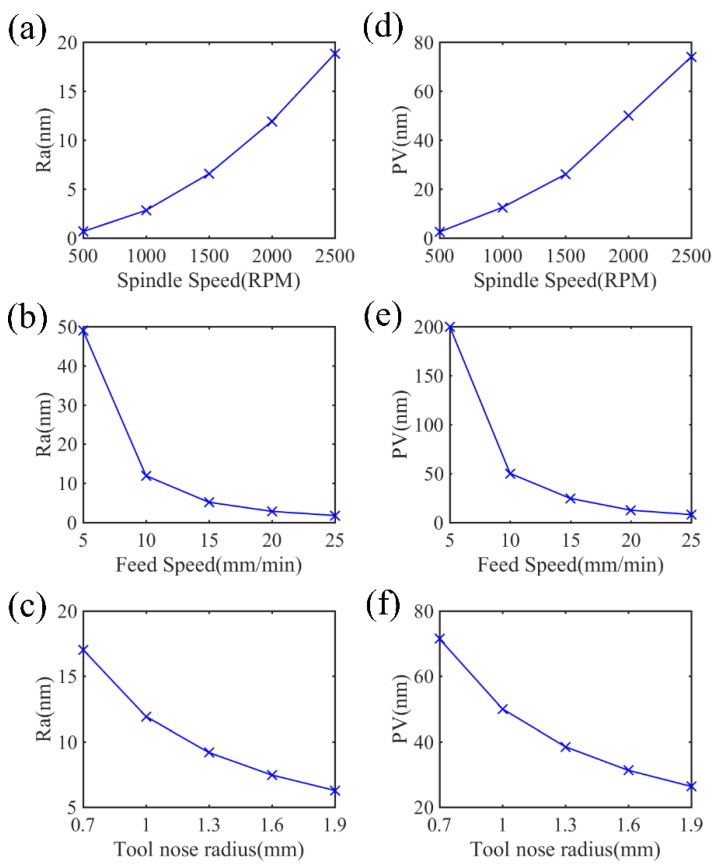
Effects of cutting parameters and the tool nose radius on the surface quality. Here, (**a**–**c**) show the trend of the surface roughness (*R_a_*) and (**d**–**f**) show the trend of the peak to valley height (*PV*) with different cutting parameters.

From the results of the simulation shown in Figure 6, it can be found that the generated 3D surface topography varies at different cutting parameters. When decreasing the feed rate and increasing the workpiece spindle speed, more intensive tool trajectories form on the machined surface, which contributes to more removed materials on the workpiece. Therefore, the surface roughness and the form accuracy drop with the rising workpiece spindle speed and the decreasing feed rate, as shown in Figure 7. In addition, the tool nose radius also bears obvious influence on the surface quality. Specifically, with increasing radius of the tool nose, the interacting area between two neighboring spiral cutting grooves is higher, resulting in less material remaining on the surface in a cutting process, so the improvement of the surface roughness and the *PV* is achieved with the growth of the tool nose radius, as shown in Figure 7c,f.

**Figure 8:**
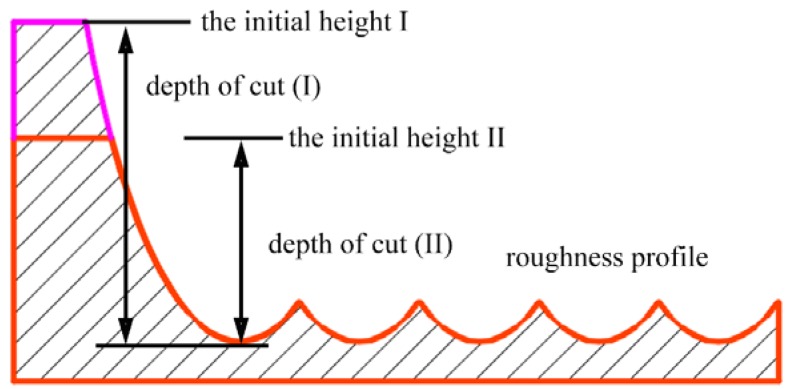
Illustration of the generated cross-sectional profile at different depths of the cut, where the cross-sectional profile under two conditions are the same when the depth of the cut is smaller than the height of the circular area of the diamond tool.

The effects of the cutting depth on the surface topography is also conducted. The results of the simulation indicate that the surface quality does not change if the depth of the cut is smaller than the height of the circular area of the diamond tool (0.65 mm), as shown in Figure 8.

**Figure 9:**
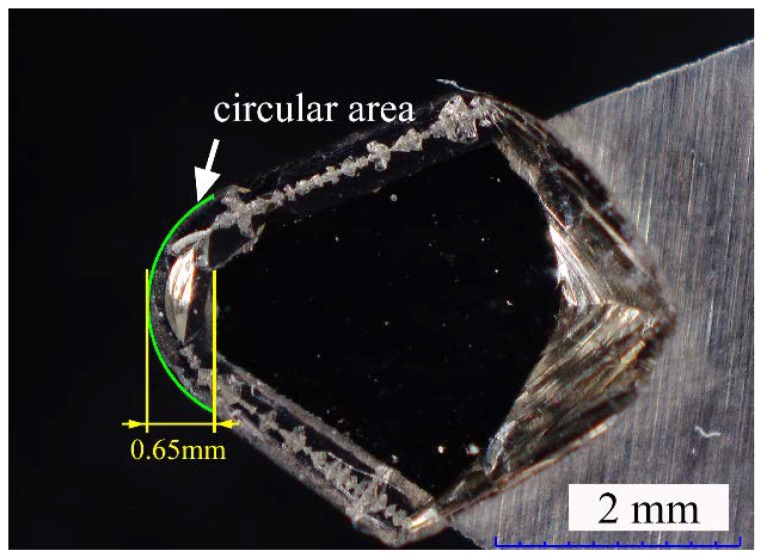
The image of the cutting edge of the diamond tool.

By measuring the cutting edge of the diamond tool, as shown in Figure 9, it can be found that the tool nose radius is around 1 mm with the height of the circular area to be about 0.65 mm, which is much higher than the selected cutting depth in single point diamond turning. Presumably, the generally used cutting depth in the micrometer range does not have an obvious influence on the surface topography.

## 3. Experiments Verification

To verify the model proposed, the experiments at selected machining parameters in ultra-precision diamond turning of the Cu alloys were performed. The element composition of Cu alloys is listed in Table 2, and the specific experimental parameters are listed in Table 3.

**Figure 10:**
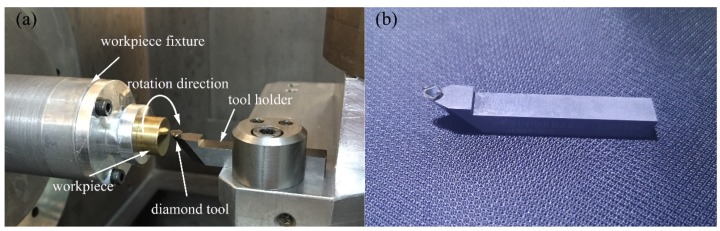
(**a**) The experimental setup and (**b**) the diamond tool.

The experiments were carried out on a four-axis ultra-precision machine, and the experimental setup and the diamond tool are shown in Figure 10. The nose radius of the used diamond tool was 1.028 mm, with the rake angle of 0°.

## 4. Results and Discussion

**Figure 11:**
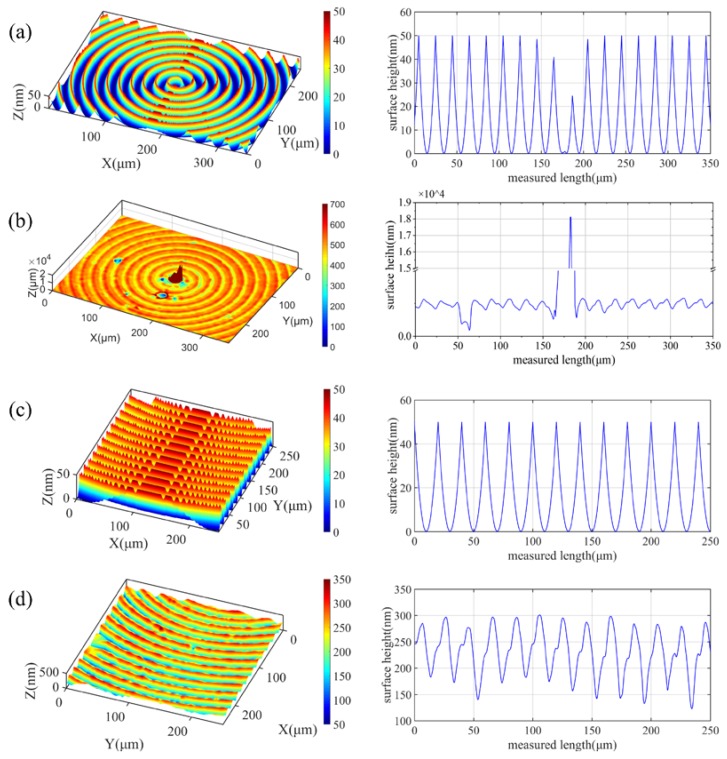
Comparison between the simulated and the machined surface at the center and at the radial distance of 0.3 mm: (**a**,**c**) are the simulated surface topography, (**b**,**d**) are the surface topography of the machined Cu workpiece.

The 3D surface topography of the machined surface was measured by a laser confocal microscopy (Sensofar S Neox 3D Optical Profiler). For comparison, the same cutting parameters and the geometry of the diamond tool in the simulation model were applied for the surface topography simulation. The same positions of the simulated and the machined surface were picked, which are shown in Figure 11. As shown in Figure 11a,b, it can be found that the center area of the machined workpiece is higher and the *PV* value of the measured center region reaches 18,127.53 nm. It is well known that the tool installation error can cause a cylinder appearance at the machined surface center in the single point diamond turning process, where the tool force pulse is formed during the formation of the center cone, and the workpiece center cone is formed by extrusion of the tool clearance face [17]. It is a phenomenon that does not occur in the simulated results as the workpiece is assumed to be ideally installed without considering any errors, as well as the elastic or plastic deformation of the Cu alloys in the simulation model. The residual profile height leads to a big difference between the simulation and the real cutting process, so further research on the surface generation mechanism at the center is necessary to effectively improve the machined surface quality.

In addition, an area of 0.3 mm away from the was chosen to compare the simulated and the experimental results, as shown in Figure 11c,d. The surface roughness *Sa* and the peak-to-valley height *PV* at varied radial distance from the center are listed in Table 4.

It can be easily found that the surface roughness and the peak-to-valley height of the machined surface are higher than the simulated results at the varied radial distance, which might be the result of the combined effects of the material microstructure, the diamond tool wear as well as the relative tool-workpiece vibration [15].

**Figure 12:**
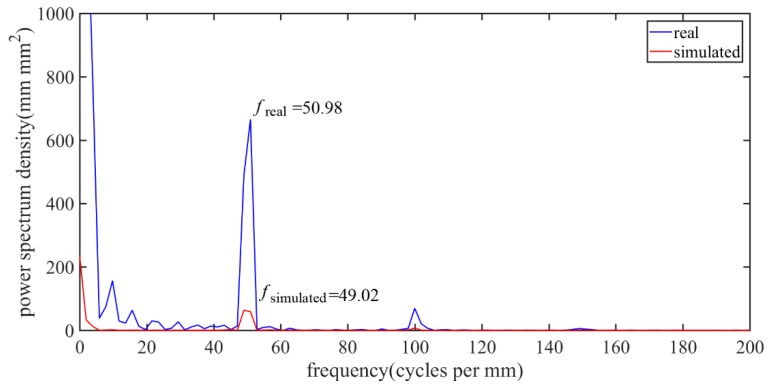
The calculated power spectrum density of the cross-sectional profile height between the simulated and the machined surface.

The calculated power spectrum density of the cross-sectional profile height data is shown in Figure 12. The specific spatial frequency of the machined surface *f*_real_ of the brass corresponds to the *f*_simulated_ from the simulated profile, which is closely related to the feed rate along the radial direction. This indicates that the trajectory in the simulated model is similar to the real cutting condition. However, the higher amplitude of the power spectrum density at 50 1/mm for the machined surface indicates a larger *PV* value, as shown in Figure 11.

### 4.1. Effects of the Tool Wear

In single point diamond turning, the diamond tool is directly involved in the cutting process, and the tool wear has multiple effects on the cutting process, including the increasing cutting force, the higher cutting temperature and the deteriorated surface quality [18]. The geometry of the diamond tool directly affects the surface topography after machining, and the surface roughness strongly depends on the sharpness and the integrity of the cutting edge [19].

**Figure 13:**
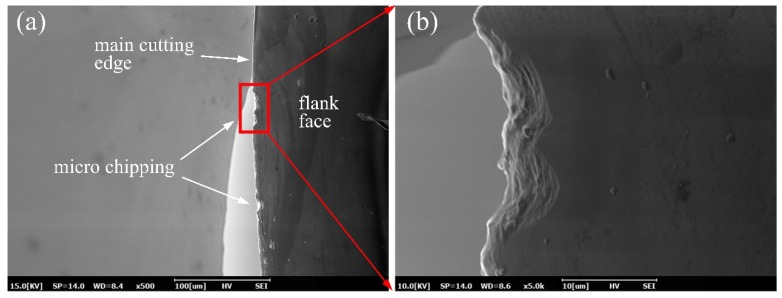
The SEM image of the diamond tool nose after cutting, where the tool wear can be found in the picture. Here, (**a**) shows that several micro-chipping appeared on the main cutting edge and (**b**) shows an enlarged area of the micro-chipping point.

**Figure 14:**
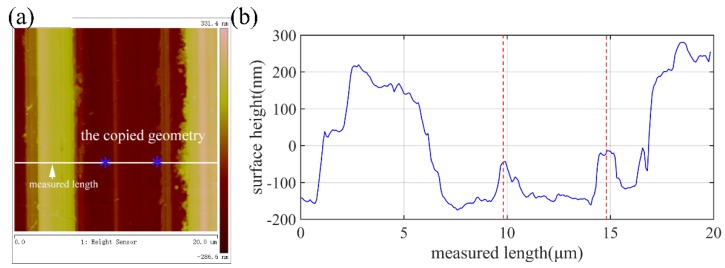
The atomic force microscope (AFM) measurement result of the machined surface. Here, (**a**) is the machined surface and (**b**) is the picked measured length.

As shown in Figure 11, the surface profile height of the experimental result is greater than the simulated surface. One of the causes is the tool wear and the effect of the built-up edge. From the SEM image of the diamond tool after the cutting experiment, as shown in Figure 13, it can be found that obvious tool wear appeared where micro-chipping occurred on the main cutting edge. When machining the copper alloys, the micro-chippings’ geometry was copied to the machined surface, as shown in Figure 14, where obvious scratches remained in the tool trajectory.

**Figure 15:**
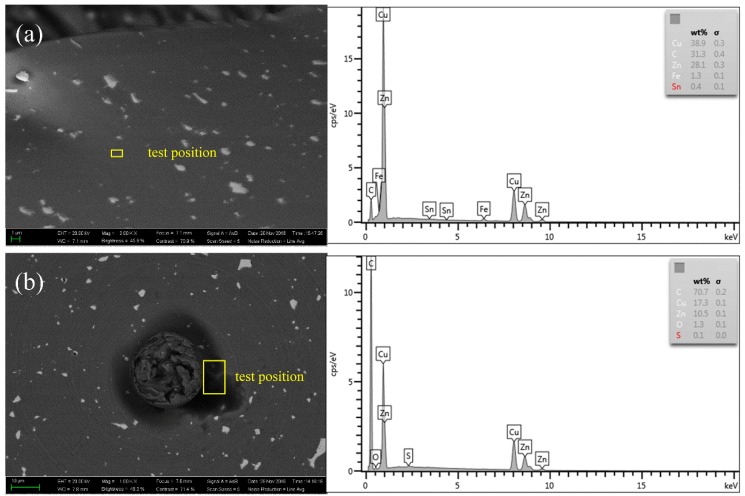
Backscattering scanning electron microscopy (BSEM) image and the EDS results of the cutting chip (**a**) and the workpiece center (**b**), where the extra carbon element is detected in the two positions.

The backscattering scanning electron microscopy image (BSEM) and the Energy Dispersive Spectroscopy (EDS) tests of the machined Cu alloys in the present study are shown in Figure 15. It can be seen that a high carbon content on both the cutting chip and the machined surface can be identified, especially at the center of the machined surface where the color of this area turns to black, as shown in Figure 15b. The extra-high carbon content on the workpiece is attributed to the graphitization of the diamond tool in the cutting process. Actually, both micro-chipping and graphitization of the diamond tool are identified in the ultra-precision diamond turning process of alloys and brittle materials [15,20,21,22,23]. The molecular dynamics simulation results indicated that graphitization occurred below the temperature point for graphitization due to the formation of active dangling bonds of diamond under the cutting force [21,22,23]. The experimental results also verified the formation of graphite from the diamond tool during the diamond turning process [15,24]. Specifically, it has been reported that the tool wear is a combined result of the cutting forces, the rising temperature and the friction [20,21]. As far as the graphitization of the surface layer of the diamond tool appeared, the abrasive wear and the micro-chipping of the diamond tool edge can be induced under the tribochemical and dynamic cutting environments in the cutting zone [25].

**Figure 16:**
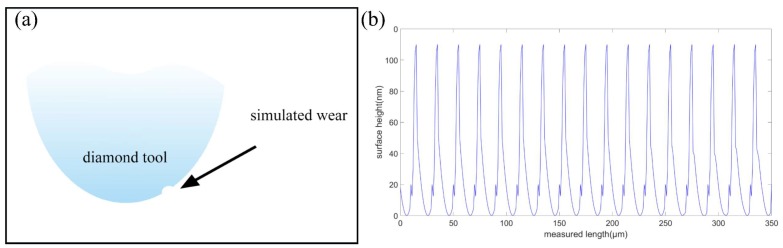
The simulated cross-sectional surface profile with tool wear taken into consideration.

Figure 16 shows the cross-sectional profile of the simulated surface with the micro-chipping on the cutting edge taken into consideration. Considering the tool wear effects, the surface topography obviously changes. The surface roughness *R_a_* and the peak-to-valley height *PV* changes to be 24.74 nm and 110 nm, respectively.

However, micro-chipping of the main cutting edge appears at many points. Both the chipping size and the chipping points were distributed randomly in the experiment. The much higher amplitude of the spatial frequency for the machined surface compared to the simulated one at around 50 1/mm also indicate multiple tool chipping. Moreover, the geometry of the cutting edge in the machining process cannot be achieved, where the influence of the tool wear on the surface topography cannot be quantitatively determined offline. Therefore, further research taking the tool wear into consideration is of great necessity to make the simulation model more accurate.

### 4.2. Effects of the Materials Microstructure

**Figure 17:**
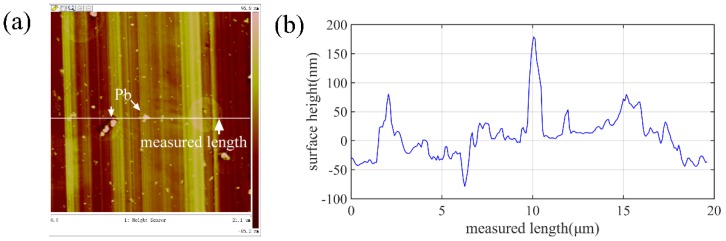
The nanoscale surface characteristics of the machined Cu measured by AFM, (**a**) surface topography of the machined surface and (**b**) the cross-sectional profile of the picked measured length.

**Figure 18:**
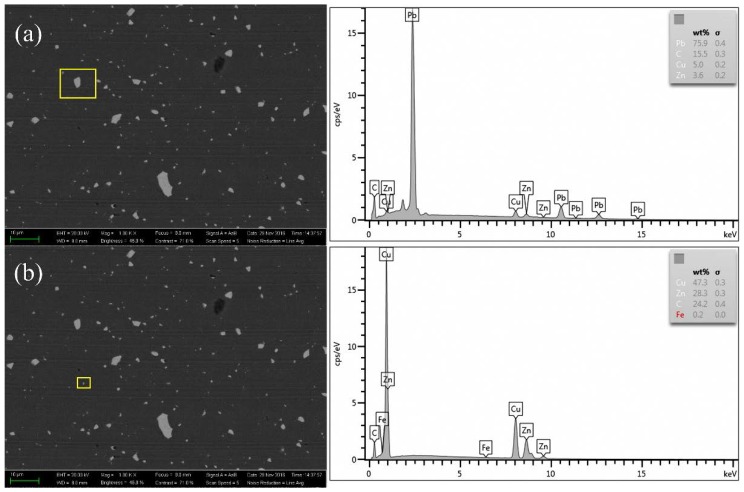
BSEM image and EDS results of the machined Cu alloys. Here, (**a**,**b**) are different test positions with different alloy element content.

Previous studies have shown that the surface topography in the ultra-precision cutting of the Cu alloys is affected by the material microstructure, including the defects [26], the crystal structure [27], the composition [28], etc. The nanoscale surface topography and the cross-sectional profile of the machined Cu alloys measured by atomic force microscope (AFM) are shown in Figure 17. The burs of random sizes and heights formed on the machined surface, as indicated by the cross-sectional profile height. The surface morphology and the composition of the elements of the randomly distributed burs are shown in Figure 18, which indicates that it is the Pb phase that contributes to formation of the surface burs, resulting in the difference between the experimental and the simulated results. In addition, many finer scratching grooves appeared in the tool feed groove. Presumably, it is induced by the multiple micro-chippings of the tool edge and the built-up edges.

## 5. Conclusions

A simulation model was established to study the influence of the cutting parameters, (workpiece spindle speed, feed rate and depth of cut), the tool nose radius, the tool wear and the material microstructure on the three-dimensional surface topography, the surface roughness and the form accuracy. The simulation results were verified by the typical experiments, which laid a theoretical foundation for revealing the surface formation mechanism of Cu alloy in ultra-precision turning. In addition, the results also showed that beyond the regular texture formed by the tool feeding, surface tears and much finer cutting scratches also formed due to the micro-chipping as a result of the graphitization of the diamond tool and the built-up edge. By comparing the simulation and experimental results, we can use the MATLAB numerical simulation method to predict the typical three-dimensional surface topography and surface roughness of Cu alloy in ultra-precision cutting. However, the effects of the elastoplastic deformation of the Cu alloy material, the surface tearing and the built-up chip edge also led to some errors between the simulation results and the experimental results, which deserves further investigation.

## Figures and Tables

**Table 1:** The cutting parameters applied in the simulation.

Set No.	Condition No.	Spindle Rotation Speed	Feed Rate	Tool Nose Radius
S1	C1	500 RPM	20 mm/min	1 mm
	C2	1000 RPM	20 mm/min	1 mm
	C3	1500 RPM	20 mm/min	1 mm
	C4	2000 RPM	20 mm/min	1 mm
	C5	2500 RPM	20 mm/min	1 mm
S2	C1	1000 RPM	5 mm/min	1 mm
	C2	1000 RPM	10 mm/min	1 mm
	C3	1000 RPM	15 mm/min	1 mm
	C4	1000 RPM	20 mm/min	1 mm
	C5	1000 RPM	25 mm/min	1 mm
S3	C1	1000 RPM	20 mm/min	0.7 mm
	C2	1000 RPM	20 mm/min	1 mm
	C3	1000 RPM	20 mm/min	1.3 mm
	C4	1000 RPM	20 mm/min	1.6 mm
	C5	1000 RPM	20 mm/min	1.9 mm

**Table 2:** The element composition of Cu alloy.

Element	Composition (%)
**Copper**	57.5–59.5
**Iron**	0.5
**Nickel**	0.5
**Plumbum**	2.0–3.0
**Zinc**	balance

**Table 3:** The selected cutting parameters for the ultra-precision turning experiments.

**Spindle speed (RPM)**	1000
**Feed rate (mm/min)**	20
**Depth of cut (μm)**	20
**Tool nose radius (mm)**	1.028
**Tool rake angle (°)**	0

**Table 4:** The surface roughness and peak-to-valley height of different areas.

Parameters	Real (Center)	Real (0.3 mm Away)	Simulated
*Sa* (nm)	49.45	39.06	12.90
*PV* (nm)	18127.53	194.23	50.00

## Notation

*f_real_*	Specific spatial frequency of the real machined surface
*f_s_*	Feed rate (mm/min)
*f_simulated_*	Specific spatial frequency of the simulated machined surface
*i*	Index for the tool tip position along the trajectory
*j*	Index for the point of the surface height profile
*PV*	Maximum peak-to-valley value (nm)
*R*	Distance from the tool tip position to the center of the workpiece
*R*_0_	Radius of the workpiece
*R_a_*	Arithmetic roughness (nm)
*rad_t_*	Tool nose radius (mm)
*Sa*	Arithmetic surface roughness (nm)
*s_s_*	Feed rate per revolution of the workpiece (mm/rev)
*t*	Machining time (s)
*X_i_*	x coordinate of the tool tip position
*Y_i_*	y coordinate of the tool tip position
*z_j_*	Height of *j*th point of the profile
*z_mean_*	Mean height of the surface profile
*θ_i_*	*i*th tool tip position of the tool in the trajectory
*ω*	Rotational speed (rad/s)
